# Irisin protects against obesity-related chronic kidney disease by regulating perirenal adipose tissue function in obese mice

**DOI:** 10.1186/s12944-022-01727-6

**Published:** 2022-11-05

**Authors:** Fang Han, Chengxia Kan, Di Wu, Zengguang Kuang, Hongwei Song, Youhong Luo, Le Zhang, Ningning Hou, Xiaodong Sun

**Affiliations:** 1 Department of Endocrinology and Metabolism, Affiliated Hospital of Weifang Medical University, 2428 Yuhe Road, Weifang, 261031 Shandong China; 2 Clinical Research Center, Affiliated Hospital of Weifang Medical University, Weifang, China; 3 Department of Pathology, Affiliated Hospital of Weifang Medical University, Weifang, China

**Keywords:** Obesity, Chronic kidney disease, Metabolic syndrome, Irisin, Adipose, therapeutic

## Abstract

**Background:**

Compared with typical visceral fat deposits in obesity and metabolic syndrome, perirenal adipose tissue (PRAT) dysfunction is more closely linked to obesity-related chronic kidney disease (OB-CKD). The myokine irisin reportedly promotes positive outcomes in metabolic disease. This study investigated whether irisin could reduce urinary albumin excretion and demonstrate renoprotective effects through the regulation of PRAT function in obese mice.

**Methods:**

C57BL/6 J mice received a high-fat diet (HFD) with or without concurrent administration of irisin. Glucose tolerance, plasma levels of free fatty acids, and urinary albumin excretion were assessed, along with renal morphology. The vascular endothelial growth factor and nitric oxide in glomeruli were also analyzed, in addition to PRAT function-associated proteins.

**Results:**

Irisin administration significantly reduced the final body weight, fat mass, and free fatty acids, without reducing PRAT mass, in HFD mice. Furthermore, irisin decreased urinary albumin excretion and attenuated both renal fibrosis and lipid accumulation. Irisin administration led to increases in PRAT function-associated proteins, including sirtuin1, uncoupling protein-1, and heme-oxygenase-1. *Ex vivo* treatment of PRAT and glomeruli with irisin also restored PRAT function. Finally, irisin treatment restored the vascular endothelial growth factor–nitric oxide axis.

**Conclusions:**

Irisin attenuated metabolic disorders and protected against OB-CKD by normalizing the PRAT–kidney axis. These results suggest that agents targeting PRAT activation might be useful for treatment of OB-CKD.

## Background

Obesity-related chronic kidney disease (OB-CKD) is receiving increasing attention because of the obesity pandemic [1–3]. OB-CKD is characterized by glomerular hypertrophy and microalbuminuria, which are also markers of systemic vascular endothelial cell lesions that involve renal arteries and glomerular endothelial cells [4, 5]. Thus, microalbuminuria links CKD to ischemic cardiovascular events [6]. Recently, research into obesity-related complications has shifted from adipose tissue distribution to the localized presence of adipose tissue around organs, including epicardial adipose tissue, perivascular adipose tissue (PVAT), and perirenal adipose tissue (PRAT) [7]. PRAT, which directly surrounds the kidneys and is closely associated with renal tissue, was originally thought to provide mechanical support alone. However, PRAT has been associated with OB-CKD [8, 9]. A previous study revealed that the presence of PRAT was predictive of microalbuminuria in obese patients [10]. PRAT can release various adipokines (e.g., adiponectin, interleukin-6, and leptin) that participate in early OB-CKD through effects on endothelial function in the renal vasculature and glomeruli [11]. The mechanism may be related to renal lipotoxicity and inflammatory infiltration. Moreover, unlike conventional visceral fat, PRAT comprises a combination of white and brown adipose tissues (WAT and BAT) [12]. PRAT can also be converted to BAT under some conditions [13]. Thus, PRAT offers a potential therapeutic target for OB-CKD.

Irisin, discovered in 2012, is a hormone secreted both by myocytes and adipose tissue [14]. Irisin can activate peroxisome proliferator-activated receptor (PPAR)α and broadly promote gene expression (e.g., uncoupling protein-1 [*UCP-1*]) in adipocytes. Irisin can also promote the phenotypic transformation of white adipocytes to brown adipocytes, thereby increasing free fatty acid (FFA) oxidation and energy consumption [15]. Because of these characteristics, irisin is presumed to have a connection with metabolic disease [16]. Elevated irisin secretion leads to increased energy expenditure, suggesting that irisin can modulate glucose homeostasis and be used for the treatment of obesity [17]. Additionally, irisin has been associated with improvements in cardiometabolic disturbances and cardiovascular disease [18]. Irisin overexpression protects vascular function by regulating endothelial and PVAT functions in obese mice [19, 20]. Patients with CKD usually have lower irisin levels, suggesting that a low level of irisin is a risk factor for CKD [21, 22]. Irisin also alleviates renal injury by improving urinary albumin [23]. However, it has been unclear whether irisin protects against OB-CKD by regulating PRAT function. The renoprotective effects of irisin in OB-CKD may be mediated by changes in PRAT function. This study investigated the impacts of irisin on urinary albumin excretion (UAE) in mice with high-fat diet (HFD)-induced obesity, then explored the mechanisms underlying its protective effects.

## Methods

### Experimental animals

Six-week-old male C57BL/6 J mice (Pengyue, Jinan, China) were assigned to three groups (*n* = 8/group). Control mice were fed a standard rodent chow, whereas HFD mice were fed an HFD (60% fat, 460 kcal/100 g) for 28 weeks. Irisin mice were fed an HFD for 28 weeks, and recombinant irisin (250 μg/kg; Phoenix Pharmaceuticals, USA) was intraperitoneally injected during the final 4 weeks. The mice were housed in standard environmentally controlled rooms and subjected to weekly measurements of body weight. Fat mass was measured using a Body Composition Analyzer (Bruker, Germany). The study protocol was approved by the Animal Ethics Committee at Weifang Medical University.

### Biochemical testing

After 28 weeks of treatment as described above, the mice were subjected to glucose tolerance and insulin sensitivity tests, as previously described [24]. Blood samples and plasma were collected. Plasma glucose and FFAs were determined by the glucose oxidase and colorimetric assays, respectively. Twenty-four-hour urine collection was performed using metabolic cages; urinary albumin and creatinine were then measured using the assay kits (Exocell Inc., USA; Jiancheng, China), respectively.

### Histology and immunohistochemistry

To evaluate morphological changes, left renal tissues were fixed in 10% formalin and embedded in paraffin. Coronal sections were collected from left kidney for histopathological examination via hematoxylin and eosin staining (Solarbio, China). To assess fibrosis, renal tissue sections were stained with picrosirius red (Sigma-Aldrich) [24]. Lipid accumulation in renal tissue sections was evaluated by Oil Red O staining [25]. Images were observed using light microscopy (magnification 400 × , Nikon, Tokyo, Japan) and analyzed by ImageJ software. Images of the cortex and medulla (3–4 images per region) from four mice in each group were analyzed.

### Preparation of PRAT-derived conditioned medium (PRAT-CM)

For *ex vivo* studies, PRAT was collected in the form of PRAT-CM, as previously described [26]. Briefly, the PRAT was separated from renal tissue, washed, and incubated at 37 °C. The PRAT-CM was then stored at -80 °C. Glomeruli were isolated, then incubated with irisin for 48 h in PRAT-CM before measurement of protein levels [27]. Vascular endothelial growth factor (VEGF) secretion from glomeruli was determined by assay kits from R&D Systems (USA).

### Western blotting analysis

Equivalent amounts of proteins from PRAT or glomeruli were homogenized, separated via sodium dodecyl sulfate–polyacrylamide gel electrophoresis, transferred to polyvinylidene fluoride membranes, and then incubated with the following antibodies: anti-glyceraldehyde-3-phosphate dehydrogenase (GAPDH), anti-heme oxygenase (HO-1), anti-sirtuin1 (SIRT1), and anti-UCP-1 (all from Cell Signaling Technology, USA), as well as anti-VEGF (Santa Cruz Biotechnology, USA). Subsequently, the blots were incubated with secondary antibody (Cell Signaling Technology, USA) and detected with Bio-Rad Laboratories (Hercules, CA, USA). Protein levels based on band intensity were quantified by ImageJ software. Band intensities were normalized to the intensity of the GAPDH band.

### Quantitative polymerase chain reaction (qPCR) analysis

Total RNA from the tissues was isolated using a Pure Link RNA mini kit (Invitrogen, USA) and reverse-transcribed to cDNA using the PrimeScript RT reagent Kit with gDNA Eraser. Primers for quantitative qPCR were as follows: tumor necrosis factor (*Tnf*)-α, forward (CCTGTAGCCCACGTCGTAG) and reverse (GGGAGTAGACAAGGTACAACCC); monocyte chemoattractant protein-1 (*Mcp-1*), forward (TAAAAACCTGGATCGGAACCAAA) and reverse (GCATTAGCTTCAGATTTACGGGT).

### Measurements of glomerular nitric oxide (NO) and mitochondrial reactive oxygen species (ROS)

Glomeruli were isolated and collected by a gradual sieving technique [27]. Total glomerular NO levels were determined by the Griess method [28]. Mitochondrial ROS production was assessed by MitoSOX Red (Invitrogen) [24].

### Statistical analysis

Data are shown as means ± standard errors of the mean; they were analyzed using GraphPad 8.0. Statistical analysis was performed by one-way or two-way analysis of variance, and interaction effects were determined by the Tukey test. The threshold of *P* < 0.05 was considered statistically significant.

## Results

### Irisin alleviated metabolic disorders in HFD mice

**Fig. 1:**
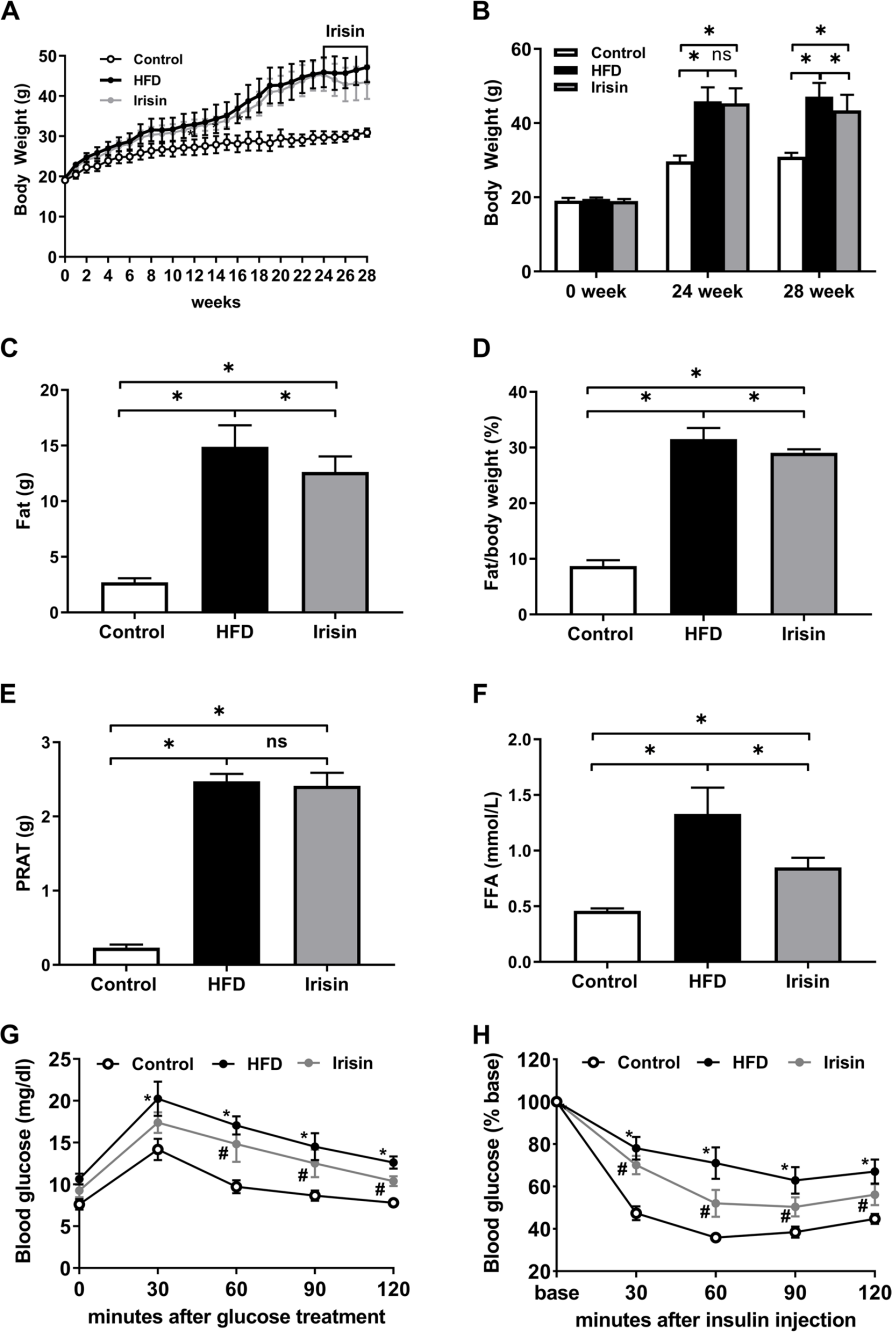
Irisin improved metabolic disorders in HFD mice. **A** body weight curve; **B** body weight; **C**, **D** fat mass; **E** PRAT; **F** FFA; **G**, **H** glucose tolerance tests and insulin sensitivity tests. ^*^P < 0.05; *n* = 5–7/group; PRAT, perirenal adipose tissue; FFA, free fatty acids

As expected, exposure to an HFD led to significant increases in body weight (1.52-fold), fat mass (5.51-fold), and PRAT mass (10.73-fold; all *P* < 0.05). Irisin treatment prevented further body weight and fat gain (*P* < 0.05), but not PRAT gain (*P* > 0.05; Fig. 1A–E). Glucose tolerance and insulin sensitivity were substantially lower in HFD mice. Irisin administration to HFD mice improved glucose homeostasis and reduced FFA levels (*P* < 0.05; Fig. 1F–H). Thus, irisin improved metabolic parameters in HFD mice.

### Irisin alleviated renal injury in HFD mice

**Fig. 2:**
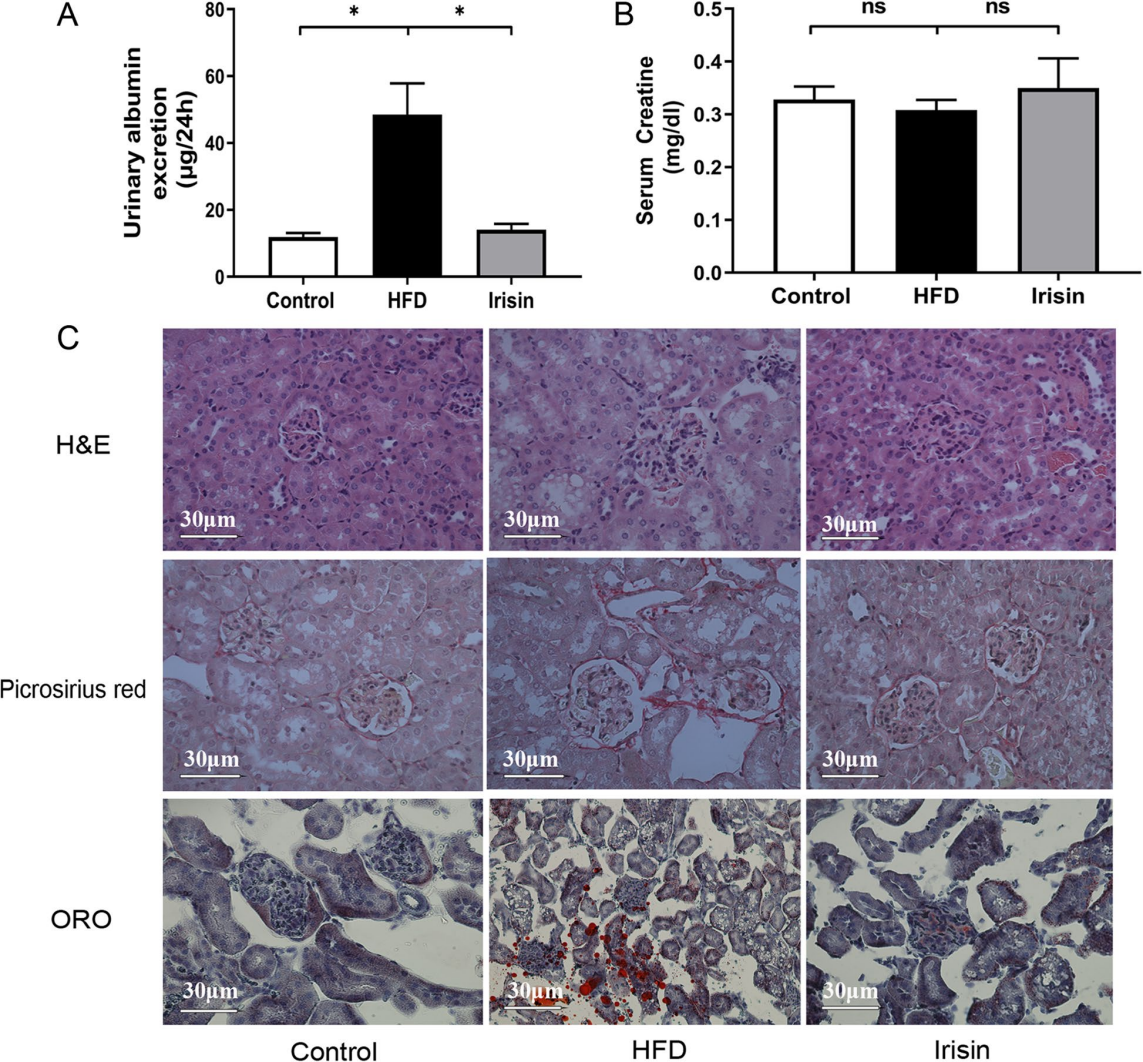
Irisin alleviated renal injury in HFD mice. **A** 24-h urinary albumin excretion; **B** albumin to creatinine ratio; **C** serum creatinine; D H&E, picrosirius red and oil red o staining; E–G quantitative analysis for glomerular area, area occupied by lipid droplets and fibrosis area. ^*^P < 0.05; *n* = 6/group for panel **A-C**; *n* = 3–4 slices from four mice/group panel E–G

Changes in renal structure and function were examined to evaluate the protective effects of irisin on OB-CKD. Twenty-four-hour UAE was substantially greater in HFD mice than in control mice (48.52 ± 22.83 µg/24 h *vs.* 11.88 ± 2.94 µg/24 h, *P* < 0.05; Fig. 2A). Compared with HFD mice, irisin mice exhibited 70.9% lower UAE (45.16 ± 10.21 µg/24 h *vs.* 14.11 ± 4.30 µg/24 h, *P* < 0.05; Fig. 2A). Similar to the UAE findings, irisin significantly reduced the albumin to creatinine ratio in HFD mice (5.31 ± 2.93 µg/µmol *vs.* 20.86 ± 10.24 µg/µmol; Fig. 2B). No significant differences were seen in creatinine (*P* > 0.05; Fig. 2C). Furthermore, HFD mice exhibited glomerular hypertrophy (glomerular area increased by 1.25-fold; *P* < 0.05; Fig. 2D, E), noticeable mesangial proliferation, glomerular fibrosis (% area increased by 92.86%; Fig. 2D, F), and significant lipid accumulation (% area increased by nine-fold; Fig. 2D, G). The abnormal pathological alterations were partially reversed by irisin administration (Fig. 2D–G). Thus, irisin alleviated HFD-induced renal injury.

### Irisin mediated kidney protection by regulating the glomerular VEGF–NO axis

**Fig. 3:**
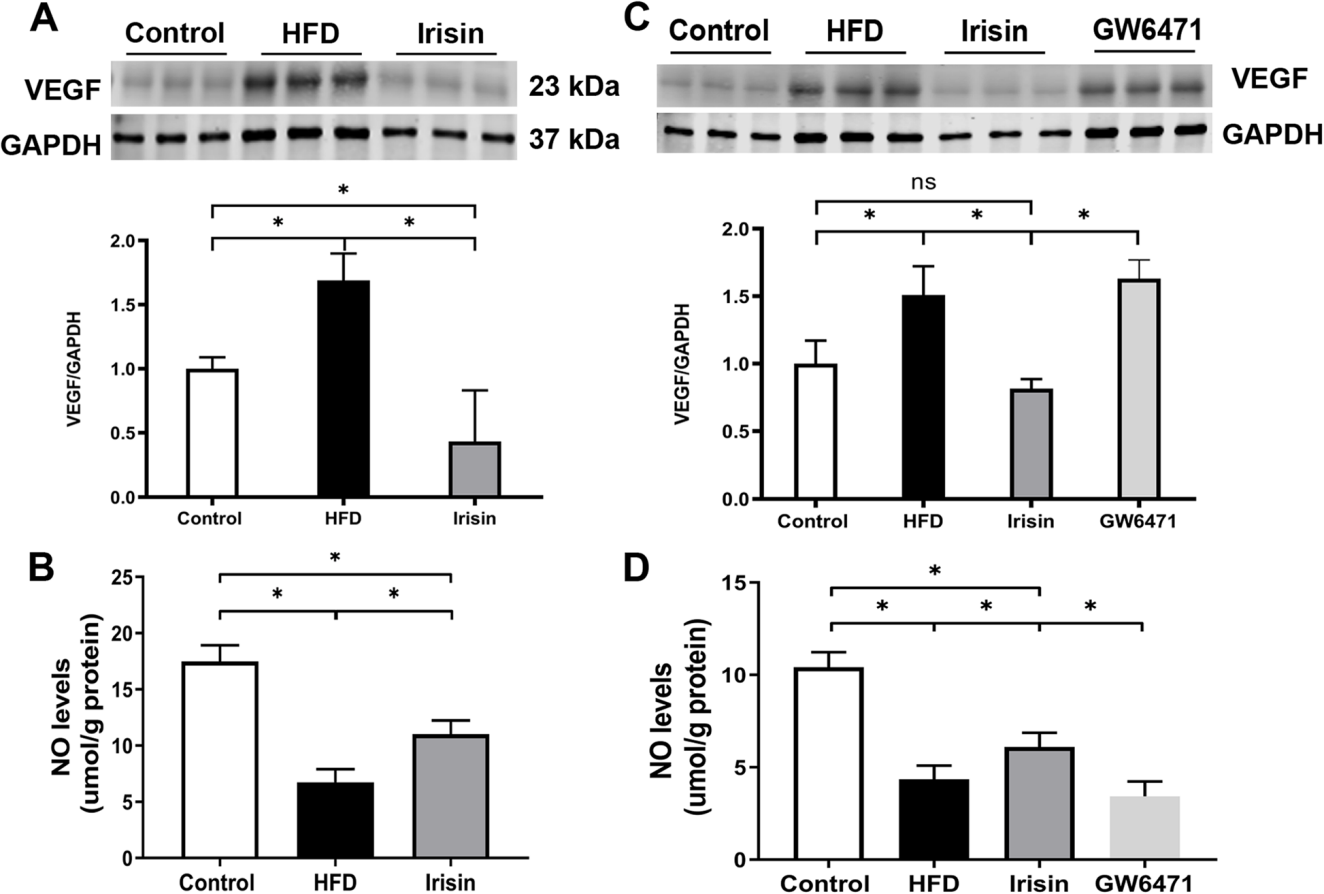
Irisin protected kidney by regulating glomerular VEGF-NO axis. **A** glomerular VEGF; **B** glomerular NO; **C**, **D** VEGF and NO levels in glomeruli pre-incubated with PRAT-CM. ^*^P < 0.05; *n* = 6/group for panel **A**, **B**; *n* = 3/group for panel **C**, **D**; VEGF, vascular endothelial growth factor; NO, nitric oxide

A previous study demonstrated that increased uncoupling of VEGF–NO axis was the main mechanism underlying the onset of OB-CKD [29]. As expected, glomerular VEGF levels were increased and NO production was reduced in HFD mice. Notably, irisin reduced VEGF levels by 74.4% and enhanced NO production by 63.8%, thus restoring the glomerular VEGF–NO axis (*P* < 0.05; Fig. 3A, B). To clarify whether irisin directly protected the PRAT-modified glomerular VEGF–NO axis, *ex vivo* studies involving PRAT-CM were performed. Treatment of glomeruli (for 48 h) with PRAT-CM collected from HFD mice led to higher VEGF levels/secretion (1762 ± 41 pg/ml *vs.*1175 ± 43 pg/ml) and lower NO production (*P* < 0.05). However, treatment with irisin partially restored normal VEGF–NO axis activity (VEGF reduction by 46.0%, VEGF secretion reduction by 38.27% with 1274 ± 41 pg/ml *vs.* 1762 ± 41 pg/ml, and NO increase by 63.8%; *P* < 0.05; Fig. 3C, D). Notably, this restoration of VEGF–NO axis activity was reversed by treatment with irisin and the PPARα inhibitor GW6471 (10 μM), which blocked irisin-mediated browning of adipocytes (Fig. 3C, D). Taken together, these findings indicate that irisin mediated kidney protection by regulating the glomerular VEGF–NO axis.

### Irisin activated UCP-1, SIRT1, and HO-1 in PRAT from HFD mice

**Fig. 4:**
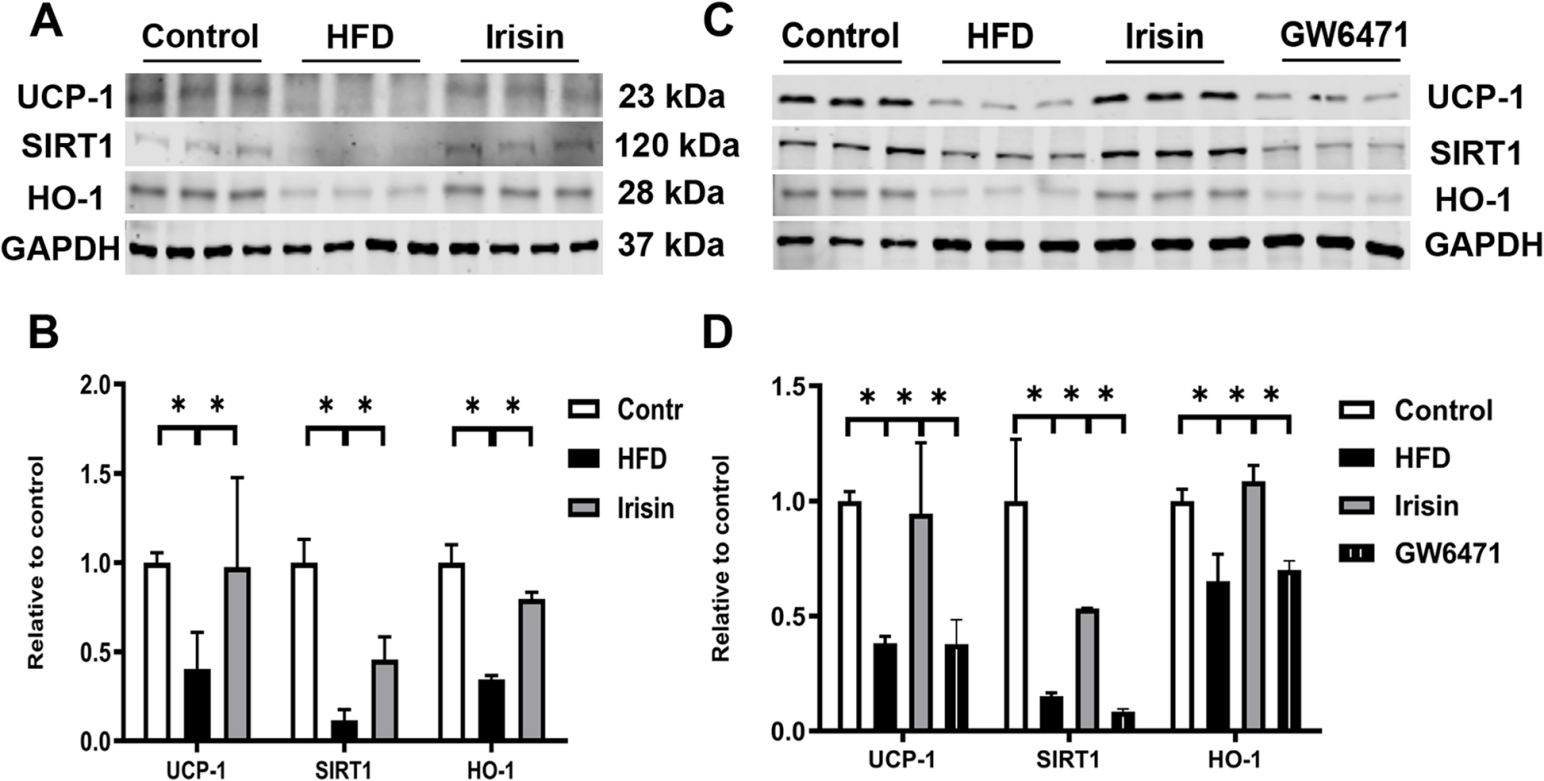
Irisin activated PRAT UCP-1, SIRT1 and HO-1 (**A**, **B**) in HFD mice *in vivo* (**A**, **B**) and *ex vivo* (**C**, **D**). ^*^P < 0.05; *n* = 6/group for panel **A**, **B**; *n* = 3/group for panel **C**, **D**; PRAT, perirenal adipose tissue; UCP-1, Uncoupling protein 1; SIRT1, sirtuin1; HO-1, heme oxygenase-1

PRAT was collected from mice to explore whether these beneficial effects on OB-CKD were associated with PRAT function. The activation of UCP-1 and the activation of SIRT1 are both associated with the browning of WAT [30]; the activation of HO-1 is associated with adipocyte reprogramming to demonstrate characteristics of browning [31]. As expected, HFD mice exhibited reduced the levels of UCP-1, SIRT1, and HO-1 in PRAT. These proteins in PRAT were enhanced after irisin administration (*P* < 0.05; Fig. 4C, D). To further investigate whether irisin directly affects these proteins, HFD mouse-derived PRAT was treated with irisin (48 h) *ex vivo*; the corresponding pathways were then evaluated in PRAT homogenates by western blotting. As expected, irisin treatment significantly activated all three pathways in PRAT (Fig. 4C, D). Similar to the findings in the previous section, these alterations were partially reversed by treatment with the PPARα inhibitor. Thus, irisin activated UCP-1, SIRT1, and HO-1 in PRAT from HFD mice.

### Irisin attenuated the production of ROS and inflammatory cytokines in PRAT

**Fig. 5:**
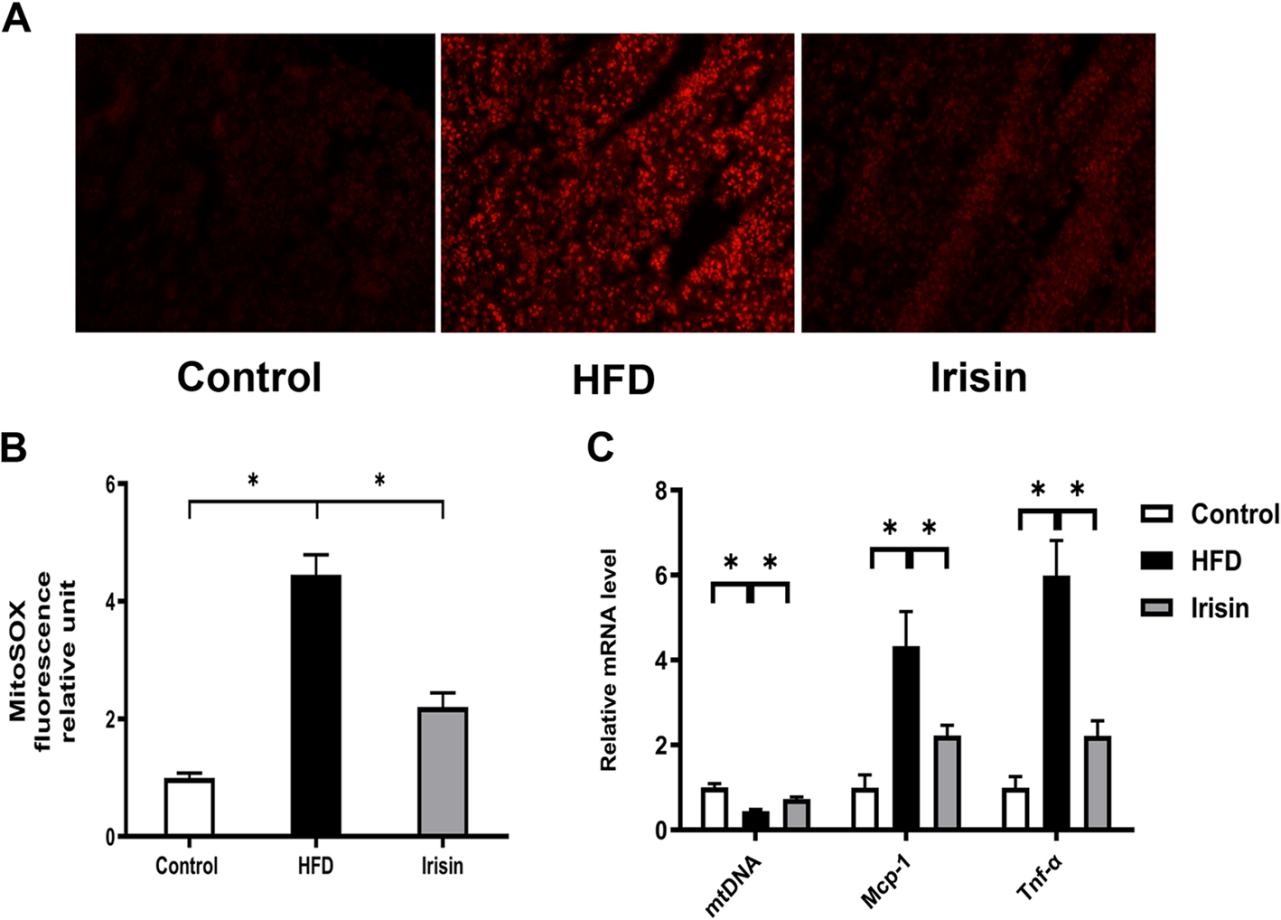
Irisin attenuated ROS and inflammatory cytokines levels in renal tissue. **A**, **B** mitochondrial ROS production, magnification × 400, fluorescent intensity was quantified by Image J; **C** mitochondrial DNA (mtDNA), *Tnf-α* and *Mcp-1* levels. *P < 0.05; *n* = 4–5/group

OB-CKD is associated with oxidative injury and inflammatory infiltration. To determine whether irisin could alleviate the production of ROS and inflammatory cytokines, mitochondrial ROS production was evaluated using the MitoSOX Red fluorescent probe. HFD mice exhibited robust production of mitochondrial ROS, *Tnf-α*, and *Mcp-1*, along with reduced mitochondrial DNA content, in renal tissue. Irisin treatment resulted in 50.57% reduction in mitochondrial ROS (*P* < 0.05; Fig. 5A, B), 62.98% reduction in *Tnf-α*, and 48.61% reduction in *Mcp-1*, as well as 64.59% enhancement of mitochondrial DNA content (*P* < 0.05; Fig. 5C).

## Discussion

The mechanisms underlying the development of OB-CKD involve lipotoxicity, enhanced oxidative stress, and the activation of various inflammatory factors. OB-CKD onset is mainly associated with the accumulation of adipose tissues, such as PRAT. This study demonstrated that irisin could reduce UAE, albumin to creatinine ratio and attenuate renal injury in HFD mice. These findings were related to the browning of PRAT and restoration of glomerular VEGF–NO axis activity. These findings indicate that the renoprotective effects of irisin in obesity are mediated by the regulation of PRAT function.

Obesity-related complications have received considerable attention in the past decade. The onset of obesity leads to metabolic alterations that promote cardiovascular and renal diseases. In this study, receipt of an HFD for 28 weeks led to obvious metabolic abnormalities, including increased body weight, fat, and FFA levels. A previous study demonstrated that PRAT is related to OB-CKD [11]. PRAT surrounds the kidneys and renal vessels; it thus has regulatory effects on renal function. PRAT accumulation can lead to increased FFA release; this induced lipotoxicity impairs renal function and exaggerates renal injury [10, 11]. In the present study, HFD promoted PRAT accumulation and enhanced the levels of circulating FFAs and renal lipids, thus amplifying renal injury in the form of increased UAE, glomerular hypertrophy, renal fibrosis, and lipid droplet accumulation.

Irisin is generated by the proteolytic processing of fibronectin type III domain-containing protein 5; this newly discovered hormone is mainly secreted by myocytes. Irisin affects energy and glucose metabolism; thus, it is potentially useful in treatments for obesity and associated metabolic alterations. Irisin may protect against cardiovascular disease [32, 33]. *Hu *et al*.* found that irisin administration significantly suppressed activation of the cardiac inflammasome, thus attenuating age-related cardiac dysfunction [32]. *Yu *et al*.* found that irisin attenuates pressure overload-induced cardiac hypertrophy, mainly by regulating adenosine monophosphate-activated protein kinase. With regard to kidney disease, circulating irisin levels have been reported to decrease with increasing CKD severity [34]. Furthermore, irisin alleviates sepsis-induced renal injury by inhibiting the NF-κB [35]. Currently, there are no published reports concerning the effects of irisin on OB-CKD. This study showed that irisin can improve metabolic abnormalities and reduce both UAE and renal injury in obese mice, indicating that irisin can protect against OB-CKD.

The effects of irisin on OB-CKD may be explained as follows. Unlike other types of CKD, OB-CKD is usually the initial step of renal injury; it principally involves vascular disorders [36, 37]. A previous study demonstrated that this early renal injury is associated with the PRAT-derived FFA-induced impaired glomerular VEGF–NO axis in obesity [11, 29]. Reduced NO production, thereby increasing VEGF production. The overproduction of VEGF could increase UAE by promoting vessel proliferation and permeability. This study showed that irisin treatment significantly increased NO production and decreased glomerular VEGF levels. Importantly, the treatment of glomeruli with HFD-derived PRAT-CM led to decreased NO production and increased VEGF levels. However, treatment with irisin restored normal VEGF–NO axis activity. These findings indicated that the PRAT-modified abnormal VEGF–NO axis participated in OB-CKD; these abnormalities could be reversed by irisin in a manner that did not require metabolic improvement.

In contrast to other visceral fat, PRAT has a “bright” characteristic and contains both BAT and WAT; it has the potential to transition from BAT into WAT. This potential was confirmed by *Efremova *et al*.*, who reported that approximately 30% of PRAT expressed UCP-1 [38]. A previous study demonstrated that irisin could promote brown-like fat in PVAT, another adipose tissue with a “bright” characteristic [20]. The present study analyzed PRAT browning to determine whether it is associated with the ability of irisin to protect against OB-CKD. Notably, treatment with irisin led to increased UCP-1 and SIRT1 in PRAT, indicating that irisin had caused browning of PRAT. Notably, this protective effect was reversed by inhibiting the browning of PRAT, as demonstrated by the ability of the PPARα inhibitor co-administered with irisin to hinder restoration of the glomerular VEGF–NO axis.

Adipose browning is associated with thermogenesis and the regulation of energy balance. This white-to-brown transdifferentiation promotes several metabolism-modifying factors; these include the anti-oxidative protein HO-1, which protects against cardiovascular disease through powerful anti-oxidative and anti-inflammatory properties [39]. Additionally, irisin-mediated vessel protection is associated with enhanced HO-1 production by PVAT. In PRAT around blood vessels and surrounding the kidneys, HO-1 production can be regulated by irisin. Activation of this pathway attenuates ROS production and inflammation; decreases in ROS production and inflammation could prevent the use of NO to form peroxynitrite, thus enhancing NO bioavailability. In the present study, treatment with irisin led to increased HO-1 production, both *in vivo* and *ex vivo*. Furthermore, after irisin treatment, the levels of mitochondrial ROS and inflammatory factors were substantially reduced. Previous studies have also shown that irisin enhances other anti-oxidative enzymes including superoxide dismutase, glutathione peroxidase, and catalase [40, 41]. These encouraging results indicate that PRAT-derived HO-1 regulates PRAT function; irisin could regulate PRAT function by enhancing the HO-1 system.

### Comparisons with other studies and what does the current work add to the existing knowledge

PRAT dysfunction has previously demonstrated a close association with OB-CKD. The present study demonstrated that irisin-mediated regulation of PRAT function could protect against OB-CKD.

### Study strengths and limitations

The strength of the study is that it highlights the potential for protecting against OB-CKD by using agents that target PRAT function. This study also had some limitations. Because irisin treatment significantly decreased body weight and fat, the irisin-related alleviation of OB-CKD may have been secondary to metabolic improvement. However, irisin also altered these protein levels in *ex vivo* experiments, indicating that irisin could protect against OB-CKD by regulating PRAT function in a manner that did not require metabolic alteration. Additionally, the delivery and distribution of irisin in PRAT and glomeruli were not investigated; the use of an irisin receptor blocker (e.g., CycloRGDyK) would provide greater support to the findings [42, 43]. Finally, PRAT function was solely monitored by changes in its mass and the levels of some essential proteins. Future PRAT activity/mass assessments should include fluorodeoxyglucose positron emission tomography/computed tomography.

## Conclusions

In summary, this study showed that the receipt of an HFD exacerbates PRAT dysfunction and kidney injury, including decreases in UCP-1/SIRT1 levels and increases in both UAE and lipid accumulation. Irisin was able to protect against OB-CKD by regulating the PRAT–kidney axis; the effects included browning of PRAT and restoration of the VEGF–NO axis. These results indicate that agents targeting PRAT activation might be useful for treatment of OB-CKD.

## Data Availability

Data will be made available on request.
